# Peculiarities of aminoacyl-tRNA synthetases from trypanosomatids

**DOI:** 10.1016/j.jbc.2021.100913

**Published:** 2021-06-25

**Authors:** Camila Parrot, Luc Moulinier, Florian Bernard, Yaser Hashem, Denis Dupuy, Marie Sissler

**Affiliations:** 1 ARNA - UMR5320 CNRS - U1212 INSERM, Université de Bordeaux, IECB, Pessac, France; 2 CSTB Complex Systems and Translational Bioinformatics, ICube laboratory and Strasbourg Federation of Translational Medicine (FMTS), CNRS, Université de Strasbourg, Strasbourg, France

**Keywords:** aminoacyl-tRNA synthetase, *trans*-splicing, kinetoplastids, species-specific insertions/extensions, mitochondria, aaRSs, aminoacyl-tRNA synthetases, CTD, C-terminal domain, MARS, multi-aaRS complex, MSA, multiple sequence alignment, mt, mitochondrial, MTS, mt targeting sequence, NTD, N-terminal domain

## Abstract

Trypanosomatid parasites are responsible for various human diseases, such as sleeping sickness, animal trypanosomiasis, or cutaneous and visceral leishmaniases. The few available drugs to fight related parasitic infections are often toxic and present poor efficiency and specificity, and thus, finding new molecular targets is imperative. Aminoacyl-tRNA synthetases (aaRSs) are essential components of the translational machinery as they catalyze the specific attachment of an amino acid onto cognate tRNA(s). In trypanosomatids, one gene encodes both cytosolic- and mitochondrial-targeted aaRSs, with only three exceptions. We identify here a unique specific feature of aaRSs from trypanosomatids, which is that most of them harbor distinct insertion and/or extension sequences. Among the 26 identified aaRSs in the trypanosome *Leishmania tarentolae*, 14 contain an additional domain or a terminal extension, confirmed in mature mRNAs by direct cDNA nanopore sequencing. Moreover, these RNA-Seq data led us to address the question of aaRS dual localization and to determine splice-site locations and the 5′-UTR lengths for each mature aaRS-encoding mRNA. Altogether, our results provided evidence for at least one specific mechanism responsible for mitochondrial addressing of some *L. tarentolae* aaRSs. We propose that these newly identified features of trypanosomatid aaRSs could be developed as relevant drug targets to combat the diseases caused by these parasites.

Aminoacyl-tRNA synthetases (aaRSs) are essential enzymes, which play a main function in mRNA translation by catalyzing the specific attachment of each amino acid onto the cognate tRNA(s). aaRSs have been partitioned into two classes (class I and class II) based on the structure of their catalytic cores and the conservation of catalytic residues: the Rossmann fold, “HIGH” motif and “KMSKS” motif for class I aaRSs and the antiparallel beta-sheet fold flanked by alpha helices with conserved motifs 1, 2, and 3 for class II aaRSs (1, 2). This partition is well preserved among species and throughout evolution. The vast majority of aaRSs also possess a tRNA anticodon-binding domain. Some of the aaRSs have additional appended structural domains as, for example, an editing domain. In higher eukaryotes, the addition during evolution of new domains has been shown to confer species-specific functional expansions beyond translation. AaRSs may thus be connected to pathways such as angiogenesis, immune response, inflammation, apoptosis, and neural development (reviewed in (3, 4, 5)). With few exceptions, there are 20 aaRSs per translating compartment of any organism.

Most eukaryotes, including the trypanosomatids (protists that belong to kinetoplastids), have at least two coding genomes: the nuclear DNA whose mRNAs are translated into the cytosol, and the mitochondrial (mt) DNA, transcribed and translated in the mitochondria. Mitochondria are the platforms of many major biochemical functions such as respiration to produce energy in the form of ATP, as well as the biosynthesis of metabolites. The mt genome has retained few coding genes: the human mt-DNA encodes 13 proteins, and the maxicircles of the kinetoplast DNA from trypanosomatids encode 18 proteins, mainly functional components of the respiratory chain complexes. The component of mt translation machinery encoded by each genome varies between species. For instance, although mt-tRNAs are transcribed from the mt-DNA in humans (6), they are all imported from the pool of cytosolic tRNAs and thus nucleus-encoded in kinetoplastids (7, 8). Mt and cytosolic aaRSs are all encoded by the nucleus, mostly by two distinct genes in humans or by a single gene in kinetoplastids. Exceptions include human LysRS and GlyRS, for which cytosolic and mt versions are encoded by a single gene and produced by either alternate splicing or alternate translation start sites, respectively (9, 10). Other exceptions are the trypanosomatid AspRSs, TrpRSs, and LysRSs that have two distinct genes encoding either the mt or the cytosolic form (11, 12, 13). In trypanosomatids, mt specialized aaRSs have been retained to accommodate tRNAs with mt -specific properties such as, for example, the mt -specific nucleotide modification of the mt-tRNA^Asp^ (11), or the mt -specific C->U RNA editing of the mt-tRNA^Trp^ anticodon to decode the mt -specific tryptophan codon UGA (12). In addition, ProRS isoforms are encoded by distinct genes in *Leishmania* species but by a single nuclear gene in *Trypanosoma* species (14). The fact that most of kinetoplastid aaRSs are dually localized (15) raises the question of mt addressing. In kinetoplastids, translatable nuclear mRNAs are obtained from polycistronic RNA precursors that are processed into mature monocistronic mRNAs by *trans*-splicing of a leader sequence to the 5′-end and polyadenylation of the 3′-end (16, 17). Alternative *trans*-splicing of the leader sequence at the 5′-end of the mRNA has been suggested to be the mechanism providing alternative translation initiation that could include or exclude an mt targeting sequence (MTS) (18, 19). This has been experimentally validated for the *Trypanosoma brucei* isoleucyl-tRNA synthetases (20).

*Leishmania* and *Trypanosoma* are two genera of streamlined protists. They belong to the class of kinetoplastida, which includes parasites that are the causative agents of various zoonotic diseases. Most parasitic kinetoplastids are heteroxenous (*i.e.*, having multiple hosts species), with different morphological forms depending on the life cycle stage. Many parasitic kinetoplastids require an insect vector for transmission. In the case of *T. brucei* species and subspecies, the parasites in the midgut of the *Glossina* fly (tsetse) are present as trypomastigote forms, while in the gut of phlebotomines (sand flies), *Leishmania* parasites transform from amastigotes (blood meal derived infected macrophages) to promastigote forms (21). Chagas disease (caused by *Trypanosoma cruzi* and transmitted by triatomine bugs) and Human African Trypanosomiasis (*Trypanosoma gambiense* and *Trypanosoma rhodesiense*) are among the most serious pathologies caused by kinetoplastids (22). Leishmaniasis is cosmopolitan in its distribution on the planet and is a serious public health problem worldwide and, more recently, to countries bordering the Mediterranean basin (*e.g.*, (23)). In addition, animal trypanosomiasis threatens millions of wild and domesticated livestock globally with important economic impacts). Development of treatments is still limited, and resistance is common (24).

Our general aim is to identify and characterize the peculiarities of molecules from the translation apparatuses in trypanosomatids with a focus here on the aaRSs. We have performed a comparative sequence analysis of aaRSs and identified and experimentally validated kinetoplastid-specific domains of aaRSs. In addition, we addressed the question of the MTS in a situation where cytosolic and mt aaRSs are encoded by a single nuclear gene. This allowed identifying new trypanosomatid-specific peculiarities that could potentially be targeted for drug design and development.

## Results

### Sequence conservation analysis

**Figure 1:**
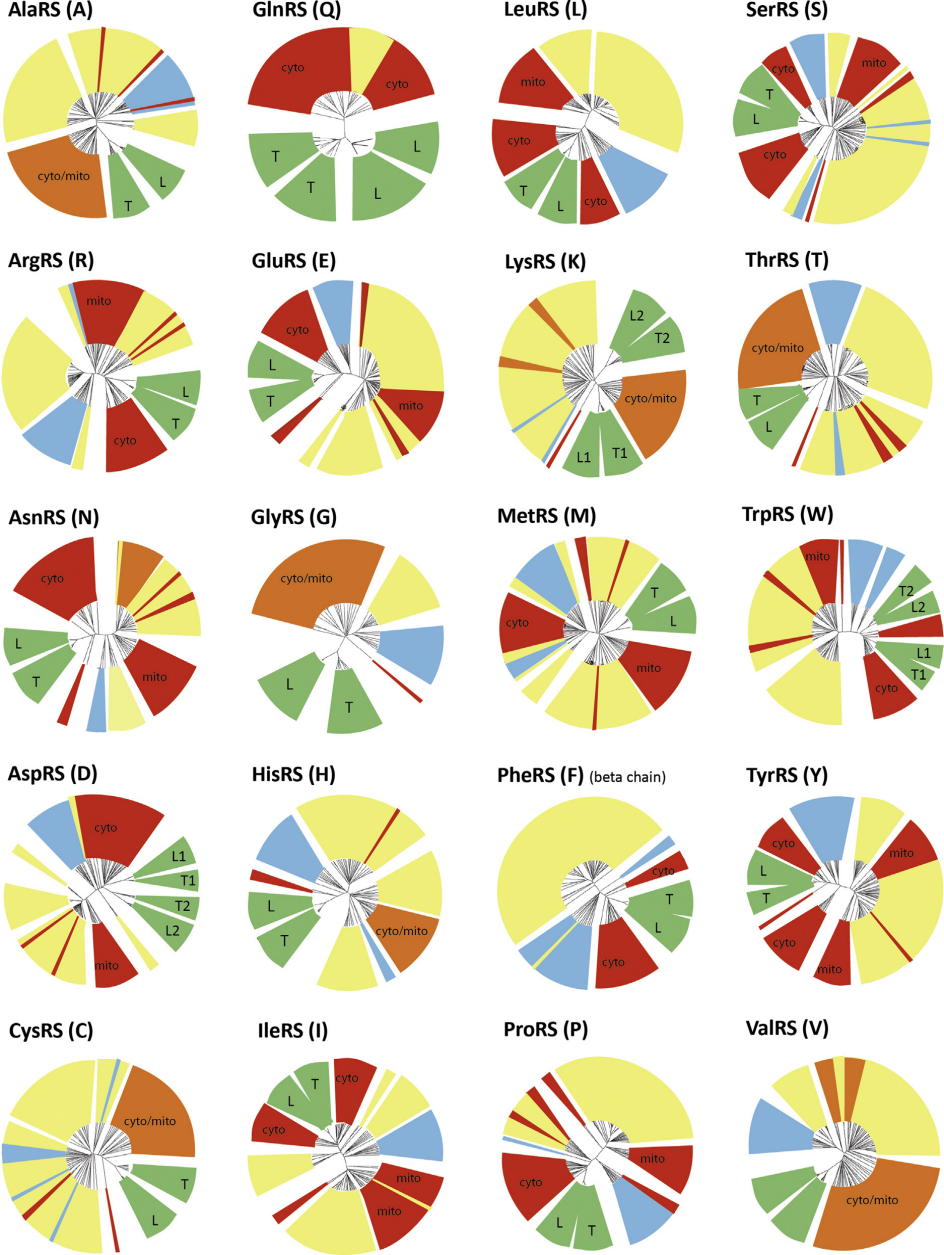
**Schematic radial representation of unrooted phylogenic trees.** Bacterial, archaeal, and eukaryotic (either cytosolic or mitochondrial) clades are colored in *yellow*, *blue*, and *red*, respectively. Monophyletic groups colored in *orange* contain indivisible mitochondrial and cytosolic sequences. The segments colored in *green* correspond to sequences from *Leishmania* and *Trypanosomatidae* species. A mitochondrial isoform is missing for GlnRS of most species that use instead the indirect pathway of transamidation of Glu-tRNA^Gln^, thanks to the presence of a nondiscriminating GluRS that recognizes both the tRNA^Glu^ and the tRNA^Gln^ (73). Protist mitochondria (including trypanosomatids and *Leishmania*), however, form Gln-tRNA^Gln^ by the direct acylation pathway (74). Original trees are given in Fig. S2.

Sequence conservation constitutes a fingerprint of selective pressure and reveals zones of functional relevance in the sequence. This conservation is analyzed by using multiple sequence alignment (MSA). For each aminoacylation system, we previously built and manually curated 3D structure–guided MSA using aaRS sequences from 92 complete genomes comprising organisms representative of bacterial, archaeal, and eukaryotic phylogenetic diversity (25). For the present study, we retrieved available aaRS sequences from 15 *Leishmania* and 12 Trypanosomatidae species from the Kinetoplastid Genomics Resources TriTrypDB (https://tritrypdb.org/tritrypdb/) (26). Newly introduced sequences are automatically aligned and clustered according to the sequence identity within the existing MSA. Unrooted phylogenetic trees based on all the aligned sequences were built and provided as radial representations (Fig. 1 and Fig. S2). Analysis of the trees indicates that in all cases but two (MetRS and mt-LysRS), trypanosomatid sequences of aaRSs clustered within cytosolic sequences from eukaryotes and archaeal sequences, revealing the eukaryotic-type origin of trypanosomatids aaRSs. This confirms existing sparse observations made with case-by-case studies (11, 12, 27) and is in agreement with the fact that all tRNAs, including the mitochondrially targeted ones, are of the eukaryotic type (7, 8).

### Identification of sequence peculiarities of trypanosomatid aaRSs

**Figure 2:**
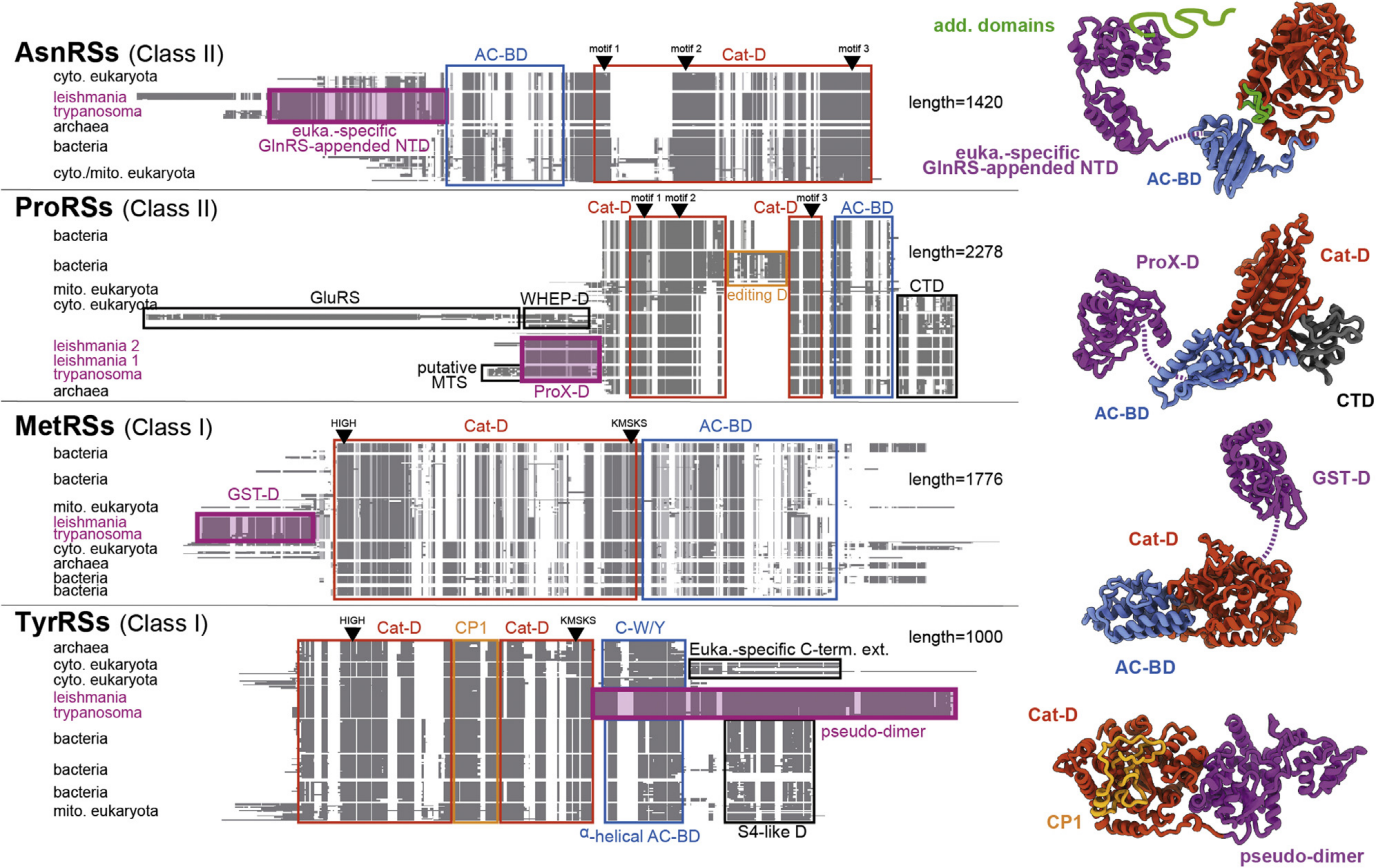
**AsnRS, ProRS, MetRS, and TyrRS harboring additional large functional domains, usually present in other aaRSs.** Schematic representation of multiple sequence alignments of aaRS protein sequences, clustered according to the phylogeny. The lengths indicated on the *right* correspond to the total number of positions for each alignment. Functional domains are *boxed* and named as follows: AC-BD; Cat-D; WHEP-D; C-W/Y; and S4-like. Catalytic residues (either HIGH and KMSKS or derivatives for class I aaRSs, or motifs 1, 2 and 3 for class II aaRSs) are positioned on the *top* of each MSA. Additional large known functional domains are *boxed* and colored in *magenta* and are GlnRS-appended NTD for the eukaryotic-specific NTD that is appended usually to GlnRS in other eukaryotic species. ProX-D stands for prokaryotic-type editing domain deacylating mischarged Cys-tRNA^Pro^ or Ala-tRNA^Pro^, GST-D for glutathione-*S*-transferase-like domain, and pseudo-dimer for the fused degenerated copy of TyrRS. Cytosolic and mitochondrial ProRSs from *L. tarentolae* have been corrected/modify according to our RNA-Seq data (see the text and Fig. S5). Homology models of *L. tarentolae* aaRS sequences are shown on the right of the alignments and serve the purpose of illustrating the positions and sizes of the additional trypanosomatid-specific domains and insertions/extensions. *Dashed purple lines* represent the link between these large additional domains (their interactions with other domains were not predicted) to other conserved domains of the aaRSs. For the sake of simplicity, all the aaRSs are displayed as monomers. Apart from the GlnRS-appended NTD, the AsnRS includes additional domains that are represented in *green thick lines*, also highlighted in Figure 3*B*. aaRSs, aminoacyl-tRNA synthetases; AC-BD, anticodon-binding domain; C-W/Y, C-terminal domain homologue to TrpRS; Cat-D, catalytic domain; MSA, multiple sequence alignment; NTD, N-terminal domain; S4-like, small RNA-binding protein S4 domain; WHEP-D, helix–turn–helix domain.

**Figure 3:**
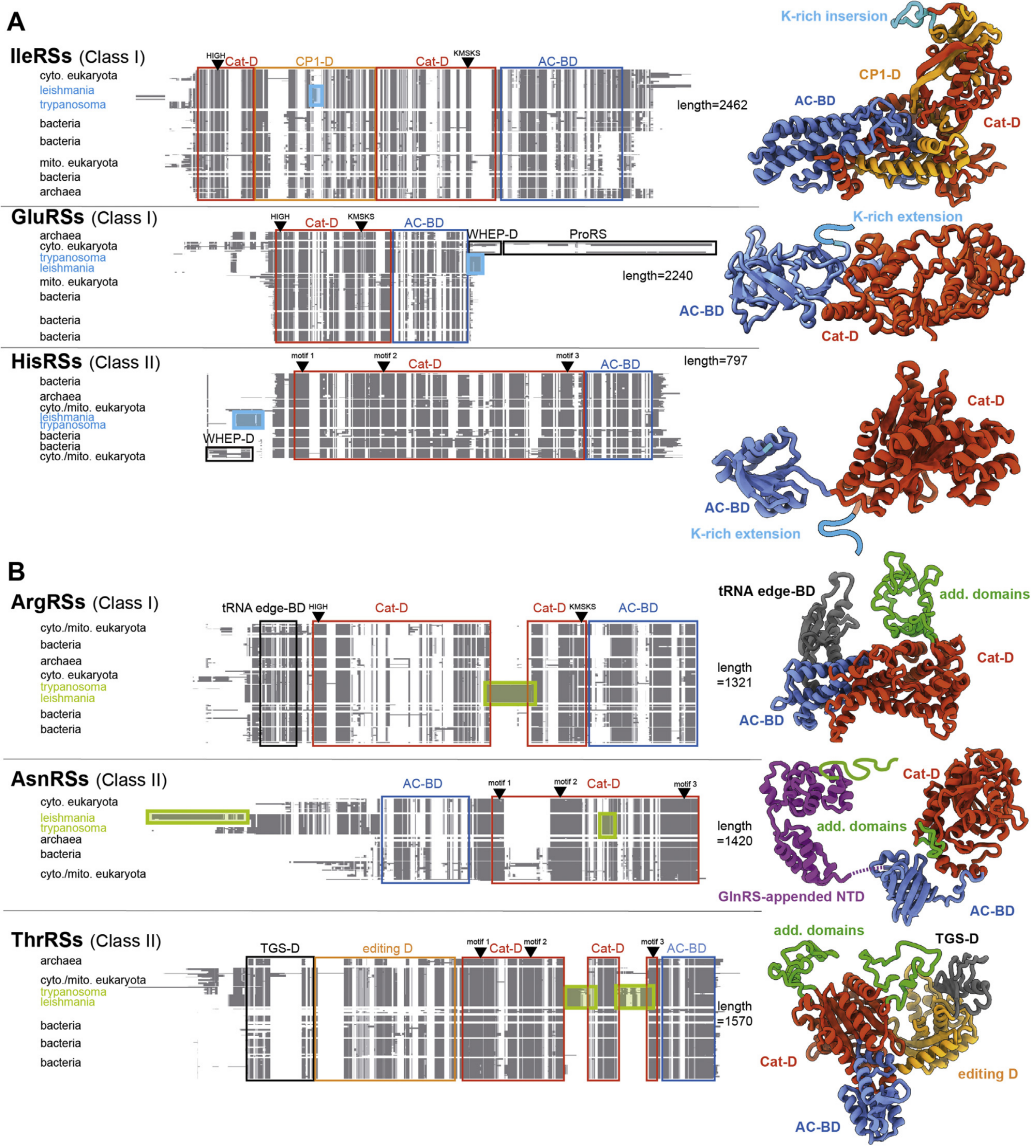
**AaRSs harboring lysine-rich insertion/extension or additional domains of unknown functions.***A*, IleRS, GluRS, and HisRS harboring a lysine-rich insertion or extension. *B*, ArgRS, AsnRS, and ThrRS with additional domains of unknown function. Schematic representation of multiple sequence alignments of aaRS protein sequences, clustered according to the phylogeny. The lengths indicated on the *right* correspond to the total number of positions for each alignment. Functional domains are *boxed* and named as follows: AC-BD; Cat-D; WHEP-D, and ProRS (which acts as an editing domain). Catalytic residues (either HIGH and KMSKS or derivatives for class I aaRSs, or motifs 1, 2, and 3 for class II aaRSs) are positioned on the top of each MSA. Lysine-rich insertion/extension or additional domains of unknown functions are *boxed* and colored in *blue* (*A*) or *green* (*B*), respectively. Homology models of *L. tarentolae* aaRS sequences are shown on the *right* of the alignments and serve the purpose of illustrating the positions and sizes of the additional trypanosomatid-specific domains and insertions/extensions. For the sake of simplicity, all the aaRSs are displayed as monomers. Besides the additional domain schematized in *green thick lines*, AsnRSs from trypanosomatids count an N-terminal GlnRS-appended domain shown in *purple*, also highlighted in Figure 2. AC-BD; anticodon-binding domain; Cat-D, catalytic domain; CP1, connective polypeptide 1; MSA, multiple sequence alignment; aaRSs, aminoacyl-tRNA synthetases; WHEP-D, helix–turn–helix domain found in TrpRS, HisRS, GluRS, and ProRS.

**Figure 4:**
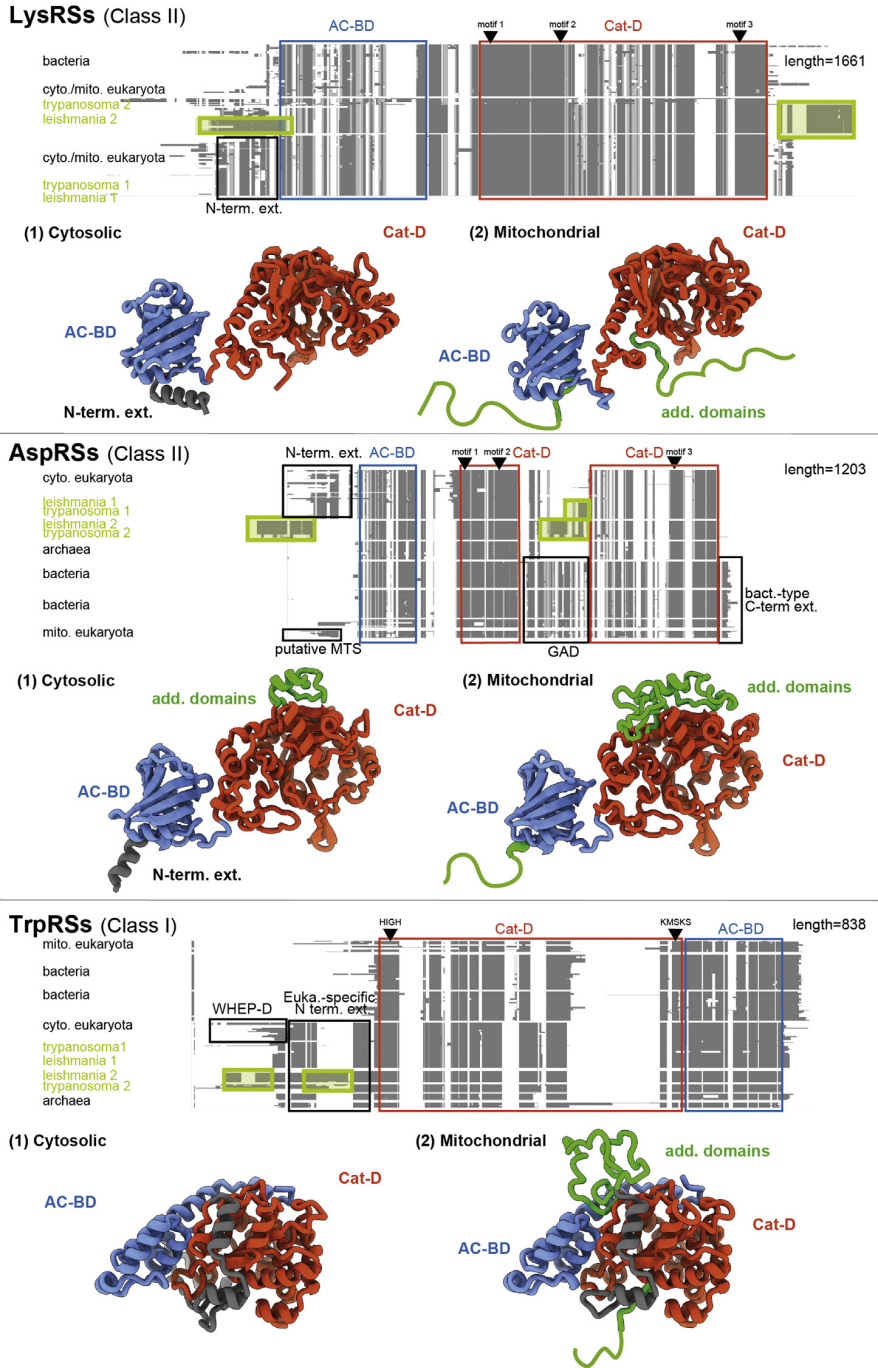
**AaRSs for which cytosolic and mitochondrial isoforms are encoded by distinct genes in trypanosomatids.** Schematic representation of multiple sequence alignments of aaRS protein sequences, clustered according to the phylogeny. The lengths indicated on the *right* correspond to the total number of positions for each alignment. Functional domains are *boxed* and named as follows: AC-BD; Cat-D; WHEP-D. Catalytic residues (either HIGH and KMSKS or derivatives for class I aaRSs, or motifs 1, 2, and 3 for class II aaRSs) are positioned on the *top* of each MSA. Additional domains of unknown functions are *boxed* and colored in *green*. Homology models of *L. tarentolae* aaRS sequences are shown below the corresponding alignments and serve the purpose of illustrating the positions and sizes of the additional trypanosomatid-specific domains and insertions/extensions (schematized in *green lines*). For the sake of simplicity, all the aaRSs are displayed as monomers. aaRSs, aminoacyl-tRNA synthetases; AC-BD, anticodon-binding domain; Cat-D, catalytic domain; MSA, multiple sequence alignment; WHEP-D, helix–turn–helix domain found in TrpRS, HisRS, GluRS, and ProRS.

For each aaRS specificity, a schematic representation of the corresponding MSA is shown (Figure 2, Figure 3, Figure 4 and Fig. S3), where structural and functional domains are annotated and further shown on corresponding homology models. Macroscopic phylum-specific sequence peculiarities are easily visible, which allows for the identification of trypanosomatid-specific insertions and extensions. Protein BLAST and SWISS-MODEL template searches were used to identify sequence and structure homologies of these insertions and extensions with existing functional domains. When no homology was found, such insertions/extensions were classified as trypanosomatid-specific domains. The analysis of the MSA thus allowed us to subdivide trypanosomatid aaRSs into four subgroups.

- The first subgroup contains eight aaRSs with no apparent characteristic feature within the alignments. This concerns the class I CysRS, GlnRS, LeuRS, ValRS and the class II AlaRS, GlyRS, SerRS, PheRS (α and β) (Fig. S3).
- The second subgroup consists of four aaRSs bearing domains that are usually reported to be appended/accessory to different aaRSs in other organisms. In trypanosomatids, these domains present divergences that are trypanosomatid specific (highlighted in purple in Fig. 2). As a first case, trypanosomatid AsnRSs have an N-terminal extension of ∼235 aa that displays sequence homology (sequence identity of ∼25% and global similarity of ∼55%) with the eukaryotic-specific GlnRS-appended N-terminal domain (NTD) (the first hit in template search in SWISS-MODEL). The GlnRS-appended NTD was demonstrated to participate in tRNA^Gln^ binding in yeast (28) and to be necessary in the human multisynthetase complex to stabilize the association of other constituents such as IleRS, MetRS, and ArgRS (29). Remarkably, this eukaryotic-specific GlnRS-appended NTD is absent from the trypanosomatid GlnRSs (Fig. S3). The second aaRSs found in this subgroup are the two isoforms (cytosolic and mt) of the ProRS, both containing a YBAK/ProX domain of ∼200 aa at the N terminus, as already observed in *Leishmania major* (14) and in *T. brucei* (30) ProRSs. This domain is a prokaryotic-type editing domain (deacylase), mostly found stand-alone and known to correct mischarged Cys-tRNA^Pro^ or Ala-tRNA^Pro^ (31). Template searches (SWISS-MODEL) highlight a higher homology with ProX, suggesting that this domain is a fused *cis*-acting editing domain that deacylates mischarged Ala-tRNA^Pro^. The third aaRS of this subgroup is MetRS. Trypanosomatid MetRSs have at their N terminus a glutathione-*S*-transferase (GST)-like domain of ∼210 aa (displaying 20% of sequence identity with the human GST and 25% with the GST-like domain appended to the human cytosolic MetRS, according to a SWISS-MODEL template search). Finally, it is known that TyrRS classically forms a homodimer and the binding site for one molecule of tRNA^Tyr^ straddles both subunits (32). In the case of trypanosomatids, our MSA confirms that TyrRS is a double-length enzyme that forms a pseudodimer, as previously observed for *L. major* and *T. brucei* TyrRSs (33, 34). The analysis of the crystallographic structure of *L. major* TyrRS revealed that the C-terminal monomer is missing some catalytic residues specific of a class I aaRS (KMSKS replaced by ARAVL), and the N-terminal monomer has a six-residue deletion that structurally affects its anticodon-binding domain (33). The same observations hold true for all trypanosomatid TyrRS sequences displayed in our MSA, which suggests that in these organisms, the pseudodimer is intrinsically asymmetric and contains only one active site and one anticodon recognition site.
- The third subgroup is composed of six aaRSs presenting insertions or extensions of completely unknown functions, for which simple BLAST or SWISS-MODEL template searches could not identify significant similarities or hits. Some of these insertions are lysine-rich and concern trypanosomatid IleRSs, GluRSs, and HisRSs, which thus display supplementary segments with ratios [number of lysines]/[length of the segment] of 25%, 23%, and 18%, respectively (highlighted in blue in Fig. 3*A*). These ratios are significantly higher than the mean frequency of lysine occurrence of 6.3% observed when considering the whole set of *Leishmania tarentolae* aaRS sequences. When looking at the whole proteome, this ratio falls back to a value closer to 3 to 5% for *Trypanosoma* and *Leishmania* species (data extracted from the codon usage database; http://www.kazusa.or.jp/codon/). Thus, a lysine-rich small domain of 24 aa is observed inserted within the editing CP1 domain IleRSS of *Leishmania* sequences (an insertion of smaller size but less conserved is yet visible in *Trypanosoma* IleRSs). As for the GluRS, a highly conserved lysine-rich C-terminal extension of ∼55 aa is found tethered to the anticodon-binding domain, and trypanosomatid HisRSs present an N-terminal lysine-rich extension of 46 aa, tethered to the catalytic domain. The remaining aaRSs of this subgroup appear more striking (Fig. 3*B*). Trypanosomatid ArgRSs have a unique insertion of ∼120 aa situated in the catalytic domain between the class I–specific catalytic motifs. This insertion is highly conserved within *Leishmania* and *Trypanosoma* sequences. Trypanosomatid AsnRSs have a well-conserved small insertion of 17 aa, situated within the catalytic core, between the catalytic motifs 2 and 3. In addition, some *Leishmania* species have an N-terminal extension of 220 aa appended to the domain similar to the eukaryotic-specific GlnRS-appended NTD (described above). Trypanosomatid ThrRSs display two insertions of ∼55 aa each. Both are situated between catalytic motifs 2 and 3. Although well conserved within each trypanosomatid lineage, these insertions display lower similarities between *Leishmania* and *Trypanosoma* (across the two lineages).
- The fourth subgroup includes aaRSs for which cytosolic and mt isoforms are encoded by distinct genes in trypanosomatids (Fig. 4). While the cytosolic LysRS has no visible sequence peculiarity, the mt isoform is characterized by two peculiar extensions: a C-terminal extension of 92 aa that is conserved among the kinetoplastid group and an N-terminal extension of 120 aa, well conserved in *Leishmania* but of smaller size and lower conservation in *Trypanosoma*. These N-terminal extensions are, however, distinct from the classical N-terminal extension of eukaryotic LysRSs. Regarding the AspRSs, both isoforms have an insertion of 33 aa for the cytosolic version and 70 aa for the mt version, which are located in place of the GAD domain of the bacterial-type AspRSs. The mt isoform has in addition an N-terminal extension of ∼130 aa in *Leishmania* that is distinct from the classical N-terminal extension of eukaryotic AspRSs. For trypanosomatid TrpRS, extra domains are visible in only the mt isoforms. These domains are, however, not conserved between *Leishmania* and *Trypanosoma*.

### RNA-Seq analysis of *L. tarentolae* aaRS transcripts

**Figure 5:**
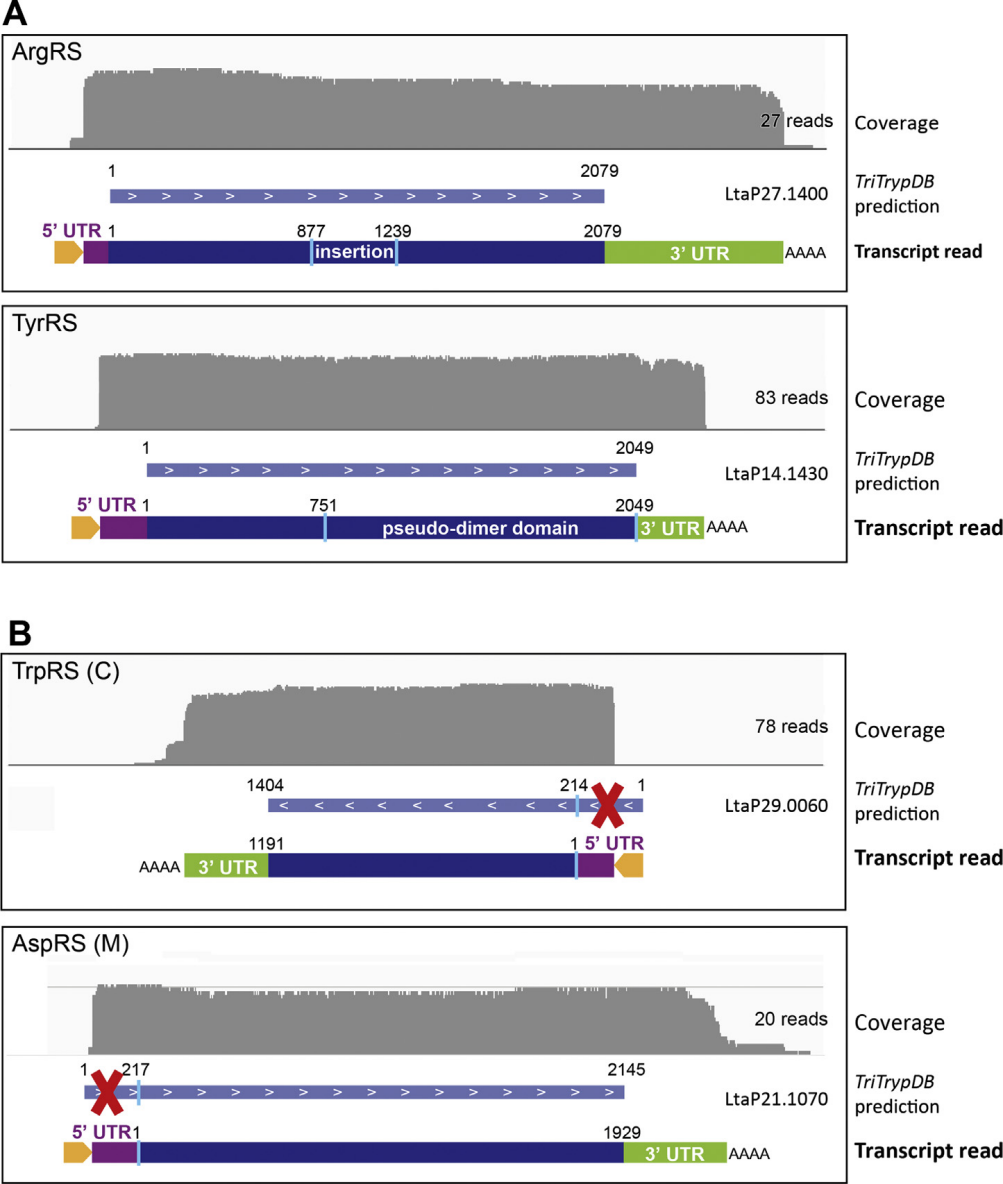
**RNA-Seq data for representative examples of *L. tarentolae* aaRSs**. The *gray panel* is a schematic representation of the coverage obtained after base calling and alignment of the reads to the reference genome (TriTrypDB-9.0_LtarentolaeParrotTarII_Genome, obtained from the TriTryp database (26)). Only reads aligning with aaRS transcripts and containing the spliced leader sequence are selected. An *orange arrow* represents the leader sequence, and the *purple* and *green boxes* represent the 5′ and 3′ UTRs, respectively. In panel *A*, trypanosomatid-specific insertion of ArgRSs and extension of TyrRSs are indicated. In panel *B*, parts of sequence missannotated in the TriTrypDB are crossed out by *red crosses*. aaRSs, aminoacyl-tRNA synthetases; C, cytosolic; M, mitochondrial.

It is established that, with a few exceptions, the RNA of trypanosomatids do not contain introns and, therefore, the genomic coding sequence reflects that of the mRNA (35). Nonetheless, mRNAs undergo two mechanisms of maturation: the addition of a spliced leader sequence (of 39 nt in *L. tarentolae*) by *trans*-splicing at the 5′-end and the polyadenylation of the 3′-end (16, 36). However, the dataset used to generate *L. tarentolae* aaRS sequences in the TriTryp database is based on genome sequencing from which gene identification was performed by homology with other *Leishmania* organisms (37). To confirm these predicted transcript sequences and to validate the presence of the above-described trypanosomatid-specific insertions and extensions within aaRS sequences, we performed direct nanopore cDNA sequencing on polyA-enriched RNA extracted from *L. tarentolae*. From the total ∼5.7 million reads, only reads containing the spliced leader sequence were selected and aligned to the genome. As shown in Table 1, not all aaRSs were sequenced with the same coverage, with numbers of reads ranking from 2 (for the IleRS) to 393 reads (for the mt-TrpRS). Nevertheless, enough reads were obtained for each aaRS to confirm that the sequences coding for the previously described trypanosomatid-specific extensions and insertions are indeed present in the mature mRNAs (selected examples are shown in Fig. 5*A* and Fig. S4).

**Table 1:** *Leishmania tarentolae* aminoacyl-tRNA synthetases: gene annotation and proposed mechanisms for the formation of the cytosolic and the mitochondrial isoforms

TriTrypDB ID	Name	MTS^a^ probability MITOPROT	Location of predicted MTS	5′-UTR size (bp) C	5′-UTR size (bp) M	Number of reads	Comments
Cytosolic and mitochondrial aaRSs encoded by two distinct genes							
LtaP30.0530	AspRS C	/	/	163	/	91	
LtaP21.1070	AspRS M	0.85	N-term	/	176	20	Start AUG misannotated in TriTrypDB
LtaP15.0260	LysRS C	/	/	89	/	65	
LtaP30.0190	LysRS M	0.99	N-term	/	168	4	
LtaP29.0060	TrpRS C	/	/	100	/	78	Start AUG misannotated in TriTrypDB
LtaP23.0360	TrpRS M	0.95	N-term	/	136	393	
LtaP29.2400	ProRS C	/	/	306	/	4	Incomplete sequence in TriTrypDB
? (LtaP18.1200)	ProRS M	0.96	N-term	/	80	8	Incomplete sequence in TriTrypDB
Cytosolic and mitochondrial aaRSs encoded a single gene							
Verified alternative *trans*-splicing^b^							
LtaP34.2190	AsnRS^c^	0.75	N-term	82	148	C61/M1	Major splice site leads to cytosolic isoform, and the alternate minor splice site leads to the mitochondrial isoform
LtaP15.1380	GlnRS^c^	0.21	N-term	139	44	C54/M1	
LtaP36.5770	IleRS^c^	0.58	N-term	229	nd	C2/M0	
LtaP30.0700	HisRS	0.64	Internal	68	27	C61/M1	
Presence of a predicted internal MTS^d^ (hypothetical mechanism)							
LtaP27.1400	ArgRS^c^	0.64	Internal	107	nd	27	Major splice site likely leads to the cytosolic isoform
LtaP11.0110	SerRS^c^	0.37	Internal	139	nd	29	
LtaP13.1000	LeuRS^c^	0.61	Internal	90	nd	18	
LtaP12.0270	CysRS^c^	0.74	Internal	259	nd	29	
LtaP22.1510	AlaRS	0.99	Internal	249	nd	28	
LtaP30.3140	ValRS	0.83	Internal	292	nd	16	
LtaP35.1480	ThrRS	0.62	Internal	526	nd	7	
LtaP36.3950	GlyRS	0.88	Internal	245	nd	29	
LtaP32.0940	PheRSα	0.99	Internal	167	nd	29	
LtaP19.0950	PheRSβ	0.91	Internal	252	nd	3	
Unpredictable mechanism							
LtaP30.3280	GluRS	0.62	N-term	?	21	92	Major splice site likely leads to the mitochondrial isoform
LtaP21.0830	MetRS	0.30	N-term	?	227	20	No alternative AUG predicted
LtaP14.1430	TyrRS	Not predicted	Not predicted	197	?	83	

Abbreviations: C, cytosolic; M mitochondrial; nd, not detected.

Sequences for *Leishmania tarentolae* aminoacyl-tRNA synthetases (aaRSs) obtained from our nanopore RNA-Seq experiments are compared with those proposed in the TriTrypDB (https://tritrypdb.org/tritrypdb/ (26)), for which the ID is listed in the left column. TriTrypDB IDs in gray correspond to sequences that are either misannotated or incomplete in the database. Corrected sequences are provided in Supporting information.

a MTS probability is predicted on the translated protein sequences by the MITOPROT software (39).

b Both alternatively spliced mRNAs were detected in our RNA-seq data with the exception of the IleRS for which only the shorter cytosolic form was sequenced.

c The same mechanism as described by Rettig *et al.* (20) or Nilsson *et al.* (18) in *Trypanosoma brucei*.

d The possibility of an internal MTS is also predicted using the MITOPROT software. No experiments were conducted to validate these predictions.

Sequencing mature *trans*-spliced mRNAs also provided information concerning splice-site location and allowed us to determine the 5′-UTR lengths (shown in Table 1), which had been completely absent from the TriTryp database. The size of the 5′-UTRs is heterogeneous among the aaRS genes ranging from 21 bp for the GluRS-encoding mRNA to 526 bp for the ThrRS-encoding mRNA.

Furthermore, precise annotation of *trans*-splicing sites enabled us to correct the misannotated sequences of some aaRSs in the TriTryp database. Thus, the cytosolic *L. tarentolae* TrpRS (LtaP29.0060) does not start at the predicted methionine but rather at the third methionine of the TriTryp proposed protein sequence, which removes 71 aa from the predicted sequences (Fig. 5*B*). Looking at the TrpRS MSA, we can assume that the *Leishmania braziliensis* cytosolic sequence (LbrM29.0070; the only other *Leishmania* sequence that display a similar N-terminal extension) is similarly misannotated, and the 78 N-terminal amino acids should be removed. Regarding the mt-AspRS (LtaP21.1070), it appears to start at the second methionine of the predicted sequence (Fig. 5*B*). This removes the first 72 amino acids from the predicted sequence. Regarding the ProRSs, two isoforms are encoded by distinct genes in *Leishmania* species (14). Full-length *L. tarentolae* ProRS sequences are however missing from the TriTryp database. Our RNA-Seq data allowed us to identify a sequence coding for the mt isoform (Fig. S5). When inserted into the ProRS MSA, it appears that the corresponding protein sequence displays an N-terminal extension of 86 amino acids, similar to the 88-aa N-terminal extension found in the *L. major* ProRS. This strongly suggests that a similar N-terminal extension (likely the MTS) is present in orthologs from other *Leishmania* species. Regarding the cytosolic isoform of the ProRS, only a fragment of 486 aa is found in the TriTrypDB (LtaP29.2400). Its insertion into the ProRS MSA showed that it corresponds to the C-terminal part of the protein but could not be a functional enzyme because catalytic residues are missing. RNA-Seq data revealed a candidate sequence, however, of incomplete reading. We manually reconstructed a consensus sequence for the *L. tarentolae* cytosolic ProRS (Fig. S5) using the very high sequence conservation observed in the alignment between the cytosolic ProRSs from trypanosomatids. It will require additional reads in RNA-Seq to be fully validated.

### One gene encodes two alternatively *trans*-spliced mRNAs

It has been proposed for *T. brucei* that alternate *trans*-splicing mechanism of an immature RNA would generate two mRNAs; one for the cytosolic isoform and one encoding an MTS for the mt isoform. This has been proposed by wide spliced leader trapping analysis (18) and experimentally validated mainly for IleRSs (20). However, the analysis of our MSA indicates that predictions made for *T. brucei* sequences do not always apply to sequences from *Leishmania species*. Indeed, alternate *trans*-splicing mechanism *a priori* requires a second in frame initiation codon AUG, upstream from the known one. Such a second AUG is not systematically conserved in trypanosomatid aaRS sequences, suggesting for instance that alternate translation initiation could occur on a codon distinct from AUG, or that some aaRSs could present an internal cryptic noncleavable MTS (*e.g.*, it was observed the *T. brucei* alternative oxidase that possesses an internal MTS in addition to the classical N-terminal and cleavable one (38)). It is also noteworthy that MTSs do not display any strict conservation of the length, sequence, or amino acid composition, rendering their identification a nontrivial task. We used the web application MITOPROT, which calculates the N-terminal protein region that can support an MTS and a cleavage site (39), as a predictive tool to identify putative mt isoforms (Table 1).

**Figure 6:**
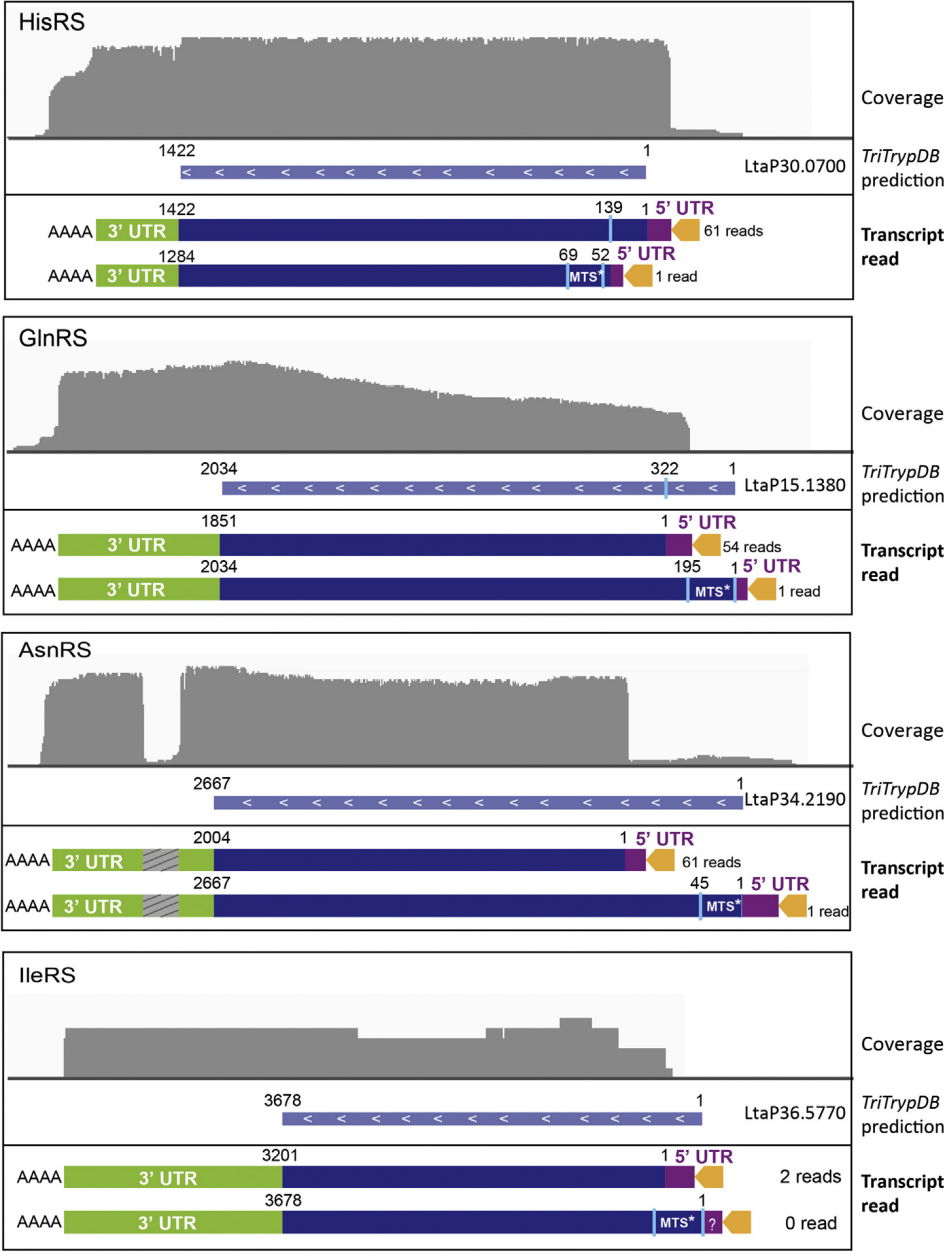
**Selected examples of alternative *trans*-splicing allowing the formation of the cytosolic and the mitochondrial isoforms of *L. tarentolae* aaRSs.** The *gray panel* is a schematic representation of the coverage obtained after base calling and alignment of the reads to the reference genome (TriTrypDB-9.0_LtarentolaeParrotTarII_Genome, obtained from the TriTryp database (26)). Only reads aligning with aaRS transcripts and containing the spliced leader sequence are selected. An *orange arrow* represents the leader sequence, and the *purple* and *green boxes* represent the 5′ and 3′ UTRs, respectively. aaRSs, aminoacyl-tRNA synthetases; C, cytosolic; M, mitochondrial; MTS, mitochondrial targeting sequence.

The analysis of our data from nanopore RNA-Seq revealed alternative *trans*-spliced mRNAs for four *L. tarentolae* aaRSs (Fig. 6 and Table 1). For both the AsnRS and the GlnRS, two transcripts were detected: a long version, encoding a predicted N-terminal MTS, corresponds to the sequence proposed in the TriTryp database, and a short version lacking the MTS concluded to encode the cytosolic isoform. In these two cases, only one read was detected for the mt isoforms, while nearly 60 reads were counted for the cytosolic ones. Two alternatively *trans*-spliced transcripts were also detected for the HisRS. However, in this case, the significantly larger number of reads obtained for the longer transcript (61 *versus* 1 for the shorter mRNA) suggests that it corresponds to the cytosolic isoform. It contains a potential internal MTS sequence, which would be concealed in the long form and exposed in the shorter transcript. Localization experiments must be carried out to confirm this hypothesis.

Finally, it is the case of IleRS, for which an alternative *trans*-splicing event has been demonstrated in *T. brucei* (20). Only two reads were counted in our data for this aaRS transcript. They are both shorter than the sequence provided by the TriTryp database and thus likely encode the cytosolic isoform, yet with a low coverage. However, a mechanism of alternate *trans*-splicing (similar to the one observed for the AsnRS and the GlnRS) remains likely because an upstream MTS can be predicted. Owing to the very low coverage of transcripts encoding the IleRS, confirmation that the sequence provided by the TriTryp (23) database corresponds to the mt isoform would require more sequencing.

## Discussion

aaRSs are ancient proteins, for which core components are well conserved across species. Nevertheless, aaRSs have specialized during evolution, which is reflected by the presence of species-specific sequence peculiarities associated with new structural and/or functional properties (*e.g.*, (5, 40, 41)) that could be used, for instance, in devising a therapeutic strategy that can discriminate pathogens from hosts. In the present study, we compare the protein sequences of aaRSs from parasitic trypanosomatids (*Leishmania* and *Trypanosoma* species) with representatives of bacterial, archaeal, and eukaryotes clades. Supported by our RNA-Seq data obtained from *L. tarentolae*, we can affirm that the majority of the trypanosomatid aaRSs have significant sequence peculiarities. These are either trypanosomatid-specific sequence extensions or insertions, for which some have no sequence similarity in other organisms, or trypanosomatid-specific arrangements of domains present in other clades.

Among domain rearrangements, the eukaryotic-specific NTD of GlnRS, known to be implicated in the stability of the multi-aaRS (MARS) complex in humans (29), is absent in the trypanosomatid GlnRSs but is appended to the AsnRSs. Interestingly, the AsnRS was not identified in the cytosolic *T. brucei* MARS complex (42) despite the presence of this NTD. Thus, it may have a distinct function than when it is fused to GlnRS. The cytosolic MARS complex from *T. brucei* was indeed partly characterized and shown to contain at least six aaRSs (MetRS, ProRS, AlaRS, TrpRS, GlnRS, and AspRS) without excluding the presence of additional enzymes, likely more transiently anchored. It has also three accessory proteins (MCP1, MCP2, and MCP3) that contain protein- and RNA-interacting domains, such as the GST domain and tRNA-binding domain, the latest contributing to enhance the tRNA-aminoacylation (42). Interestingly, a GST-like protein–protein interaction motif is found appended to MetRS from trypanosomatids. The GST-like domain is commonly found in eukaryotic GluRS, ValRS, CysRS, and MetRS but was shown to be mostly absent from lower eukaryote aaRSs, and entirely absent in prokaryotes (43). As shown in our MSA, it is missing in eukaryotic-type ValRS, CysRS, and GluRS from trypanosomatids but found singularly appended to the bacterial-type MetRS. Another peculiar domain rearrangement concerns the ProRSs. While Ybak/ProX domains are known as independent *trans*-acting deacylases that edit mis-aminoacylated tRNAs (31), a ProX domain is found uniquely appended to both cytosolic and mt isoforms of ProRS in trypanosomatids. It is thus likely a *cis*-acting editing domain that deacylates mischarged Ala-tRNA^Pro^. Noteworthy, two freestanding Ybak domains were additionally found as stand-alone proteins in *Leishmania major* (14).

We also identified trypanosomatid-specific insertions and extensions within aaRS sequences, for which simple sequence comparison could not highlight a known function and for which the role or property remains to be identified. Among them, some are lysine-rich domains and found within trypanosomatid IleRSs, GluRSs, and HisRSs. The role of lysine-rich extensions has been investigated in the context of aaRS function, and it has been demonstrated, for instance, to be important for tRNA binding in several different organisms (44, 45, 46). More investigations could verify if the trypanosomatid-specific lysine-rich extensions have a similar function. Regarding the insertions and extensions identified within trypanosomatid ArgRSs, AsnRSs, ThrRSs, LysRS, AspRSs, and TrpRSs, little is known about their possible function(s). To our knowledge, only the role of the C-terminal extension of mt LysRSs has been deciphered (13). Indeed, it was demonstrated that this extension is used to block the enzyme activity as long as the protein is present in the cytosol, preventing any detrimental interaction between cytosolic and mt translation apparatuses (13). This domain is cleaved once the protein is present in the mitochondria, which enables the activation of the mt LysRS (13). We performed 3D modeling of the *L. tarentolae* aaRSs. Our homology models serve the purpose of illustrating the positions of the trypanosomatid-specific additional insertions/extensions/domains that may be often misrepresented. However, the models show that all of these extra pieces of sequences are at positions likely accessible to permit new domains to occur (mainly Fig. 3*B*). Many of them have no significant sequence similarity with known functional domain, but they are all situated on a face of the protein that is distinct from the tRNA-interacting surface, further suggesting that these extra domains could contribute to trypanosomatid-specific functions and properties that will require deeper investigation.

Finally, it is worth recalling that in trypanosomatids, cytosolic and mt isoforms of aaRSs are mainly encoded by a single nuclear gene. Although it has been shown that each of these aaRSs is indeed required in the mitochondria and therefore imported into it (15, 47), the way the two isoforms (in particular the mt ones) are produced remains poorly understood. It has been proposed for *T. brucei* that alternative *trans*-splicing mechanism of an immature RNA would generate two mRNAs; one for the cytosolic isoform and one encoding an MTS for the mt isoform (18, 20). It is unknown whether the mechanism is identical in *Trypanosoma* and *Leishmania* species, but our MSA already suggest that some differences can exist because, for instance, alternative starts are poorly conserved among trypanosomatids. As for *T. brucei* (18, 20), our RNA-Seq data revealed that four of the aaRS-encoding mRNAs of *L. tarentolae* indeed undergo alternative *trans*-splicing leading to the production of the two isoforms. However, the weak coverage that we observed for the mRNA transcripts corresponding to the aaRS mt isoforms demonstrate that detecting dually targeted protein through alternative *trans*-splicing may be challenging. In trypanosomatids, only 18 proteins are translated in the mitochondria, making the need for aminoacylated-tRNAs substantially lesser in the mitochondria than in the cytosol (48). Of course, the same holds true for most other eukaryotic species. For other aaRSs with no detected alternative *trans*-splicing, the question of mt targeting remains open. However, several hypotheses could be proposed, in addition to the simple possibility that the alternative *trans*-splicing event occurs too rarely to be detected by RNA-Seq. For nine of the remaining 16 aaRSs, an internal MTS is predicted by the MITOPROT web server (39) with good probabilities (Table 1). It therefore cannot be excluded that rare alternative *trans*-splicing events could occur, leading to a situation similar to what we already observed for the HisRSs: the long mRNA corresponding to the cytosolic isoform, and a short mRNA, in which the concealed MTS is unveiled, corresponding to the mt isoform. Another possibility is that only one isoform is produced that contains a cryptic internal MTS, allowing for part of the cytosolic protein pool to be transported to the mitochondria (38). Finally, for some aaRSs, no model could be proposed for several reasons; no MTS sequence was predicted, no alternative *trans*-splicing event was detected, or no alternative AUG was found (Table 1). Thus, more research should be done to decipher how some of these enzymes are targeted to each compartment.

To conclude, the present study clearly shows that aaRSs from parasitic trypanosomatids possess unique sequence peculiarities that distinguish them from their orthologs in other organisms, including their possible hosts. Future investigations will shed light on the functional relevance of these sequences and the cellular properties they confer. Of note, aaRSs have been designated as potential drug targets at several instances for their sensitivity to known catalytic inhibitors (49). For instance, the antimicrobial Borrelidin, a known inhibitor of ThrRS, was shown to inhibit *T. brucei* or *Leishmania donovani* ThrRS (49, 50), leading to antitrypanosomal activity. Structure-based studies have allowed the discovery of inhibitory compounds of *L. major* or *T. brucei* MetRSs (51, 52). In another study, predictive computational tRNA network analyses combined to biochemical validation suggest that parasite-specific tRNA–aaRS interactions are sufficiently divergent from homologous human machinery to be potential molecular targets (53). Nevertheless, the abovementioned studies propose to target the canonical aminoacylation function. To the best of our knowledge, the trypanosomatid-specific insertions/extensions, situated for most of them on a face of the protein that is distinct from the tRNA-interacting surface, have never been considered in any future therapeutic strategy. Therefore, the identification of such trypanosomatid-specific sequence specificities, potentially independent from their canonical function, may open the path for the development of novel therapeutic applications at a time when parasitic trypanosomatids, causative of various zoonotic diseases, are increasingly spreading (23, 54).

## Experimental procedures

### Sequence retrieval

AaRS protein and genomic sequences from trypanosomatids (15 *Leishmania* and 12 *Trypanosoma* species) were retrieved, when available, from the TriTryp database (https://tritrypdb.org/tritrypdb/ (26)). The list of the organisms is provided in Figure S1.

### Sequence alignments

The aaRS core MSA were previously created (25). These alignments (one per aminoacylation system) are constituted of aaRS sequences from 92 organisms, which are representative of bacterial, archaeal, and eukaryotic phylogenetic diversity. For the present study, we introduced aaRS protein sequences from *Leishmania* and *Trypanosoma* species (the list of the organisms is provided in Fig. S1). Newly introduced sequences are individually aligned against the core alignment using the add option of the MAFFT program (55). The alignments are subsequently clustered according to sequence identity using the DPC (Density Point Clustering) program (56), and the number of clusters is determined using the Bayesian Information Criterion (57). Unrooted phylogenetic trees based on all the aligned sequences are built using the FastME program (58). Schematic representations of the alignments are obtained by replacing residues by gray pixels over a white background, which allows easily emphasizing sequence-specific insertions or extensions. Finally, the alignments were stored in the MACSIMS XML format (59). All the abovementioned alignment manipulations (sequence addition, edition, clustering, phylogenetic tree building, schematic overviews) were done inside the workbench ORDALIE (ORDered ALIgnment Information Explorer), specifically dedicated to the analysis and exploration of the informational content of an MSA (L. Moulinier, unpublished results).

### Cells

*L. tarentolae* (ATCC PRA229) were grown at 26 °C with agitation in the brain heart infusion medium containing 10 μg/ml hemin and supplemented with 10% fetal bovine serum to the late log phase.

### RNA extraction and nanopore sequencing

*L. tarentolae* cells were resuspended in 1 ml of RNABLE with 10% chloroform and lysed by three freeze-thaw cycles. Centrifugation was performed for 10 min at 12,000*g* at 4 °C. The upper phase was collected, and nucleic acids were precipitated overnight at 4 °C with one volume of isopropanol. After centrifugation for 10 min at 12,000*g* at 4 °C, the pellet was washed with 70% ethanol and resuspended in nuclease-free water. DNase treatment was performed according to manufacturers' instructions (TURBO DNA-free, AM1907, Invitrogen). Starting from 75 μg of total RNA, poly-A mRNAs were enriched using poly(dT) magnetic beads (Dynabeads Oligo(dT)25, 61005, Invitrogen). Retro-transcription, sample preparation, and cDNA sequencing were performed according to nanopore Direct cDNA Sequencing instructions (SQK-DCS109). The obtained cDNA library was loaded and sequenced on MinION R9.4.1 flow cells (Oxford Nanopore Technologies) for 48 h. Base calling was performed using Guppy basecaller ONT (Oxford Nanopore Technologies). Reads were aligned to the reference genome (TriTrypDB-9.0_LtarentolaeParrotTarII_Genome, obtained from the TriTryp database (26)) using minimap2 (60) and visualized using IGV tool (61). In house–made python scripts were developed to select only reads aligning with aaRS transcripts and containing the spliced leader sequence. The script has been deposited in the GitHub database https://github.com/FlorianBrnrd/aars-nanopore-pipeline.

### Three-dimensional modeling

The extracted *L. tarentolae* aaRSs sequences from the TriTryp database were used to generate homology-based models using the online web service SWISS-MODEL (62). The used templates were selected based on their homology scores to the query sequences and the resolutions of their structures. The produced homology models only serve the purpose of illustrating the positions and sizes of the additional trypanosomatid-specific domains and insertions/extensions. For group 2 aaRSs, templates 5xix (63) and 4ye8 (64) were used to model the C-terminal domain (CTD) and NTD of AsnRS, 6swx (51) and 4kf9 to model the CTD and NTD of MetRS, 4hvc (65) and 1vki to model the CTD and NTD of ProRS, and the TyrRS X-ray structure (3p0j) was already solved from *L. major* (33) and used as a template for *L.* *tarentolae* corresponding TyrRS. For group 3 aaRSs, template 4q2t (66) was used to model the ArgRS, 5bnz for the GluRS, 4yrf (67) for the HisRS, 6ldk (68) for the IleRS, and 1nyq (69) for the ThrRS. Finally, for group 4 aaRSs, template 6od8 was used to model both the cytosolic and mt isoforms of the AspRS, templates 6bni and 6ilh were used to model the cytosolic and mt isoforms of the LysRS, respectively, and templates 5ujj (70) and 1r6t (71) to model the cytosolic and mt isoforms of the TrpRS, respectively. All homology models’ figures were rendered in UCSF ChimeraX (72).

## Data availability

The data that support the findings of this study are contained within the article and the supporting information. All source data generated for this study are available from the corresponding author (Dr Marie Sissler; m.sissler@iecb.u-bordeaux.fr) upon reasonable request.

## Supporting information

This article contains supporting information.

## Conflict of interest

The authors declare that they have no conflicts of interest with the contents of this article.
