# Supplementary material for: Peculiarities of aminoacyl-tRNA synthetases from trypanosomatids
Source: J Biol Chem. 2021 Jun 25;297(2):100913. doi: 10.1016/j.jbc.2021.100913 (PMC8319005; doi:10.1016/j.jbc.2021.100913)
Supplement: Supplemental Figures S1–S5 [file mmc1.pdf]

Supporting Figure S1. List of organisms available in the tritrypDB (https://tritrypdb.org/)

	Taxonomy ID
**Leishmania**	
L. aethiopica	5667
L. amazonensis	5659
L. arabica	40284
L. braziliensis	5660
L. donovani	5661
L. enriettii	5663
L. gerbilli	40285
L. infantum	5671
L. major	5664
L. mexicana	5665
L. panamensis	5679
L. tarentolae	5689
L. tropica	5666
L. turanica	62297
**Trypanosoma**	
T. brucei	5691
T. congolense	5692
T. cruzi	5693
T. evansi	5697
T. grayi	71804
T. rangeli	5698
T. theileri	67003
T. vivax	5699

Supporting Figure S2. Radial representation of unrooted phylogenic trees. The trees are built using the FastME program (32). Sequences in yellow, blue and red are from bacteria, archae, and eukaryotes (cytosolic or mitochondrial), respectively.

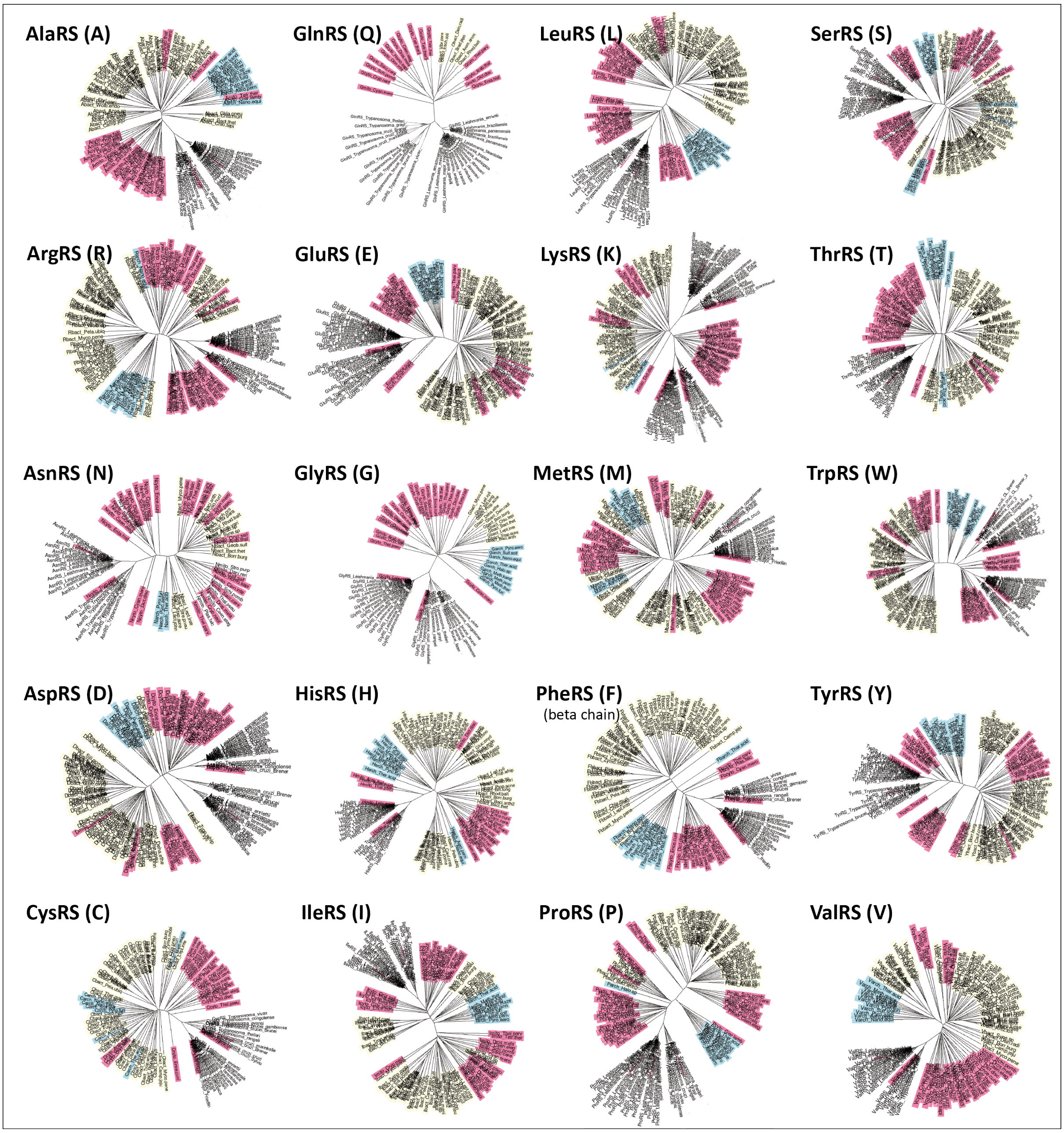

Supporting Figure S3. Schematic representation of downsized multiple sequence alignments of aaRS protein sequences that are clustered according to the phylogeny. The lengths indicated on the right correspond to the total number of positions for each alignment. Functional domains are boxed and named as followed: AC-BD stands for anticodon-binding domain; Cat-D for catalytic domain; WHEP-D refers to the helix-turn-helix domain found in TrpRS, HisRS, GluRS and ProRS; CP1 stands for Connective Polypeptide 1 and acts as an editing domain. tRNA-edge BD and tRNA-DB stand for tRNA-edge binding domain and tRNA binding domain, respectively. Catalytic residues (either HIGH and KMSKS or derivatives for class I aaRSs, or motifs 1, 2 and 3 for class II aaRSs) are positioned on the top of each MSA.

Supplementary Figure S3.

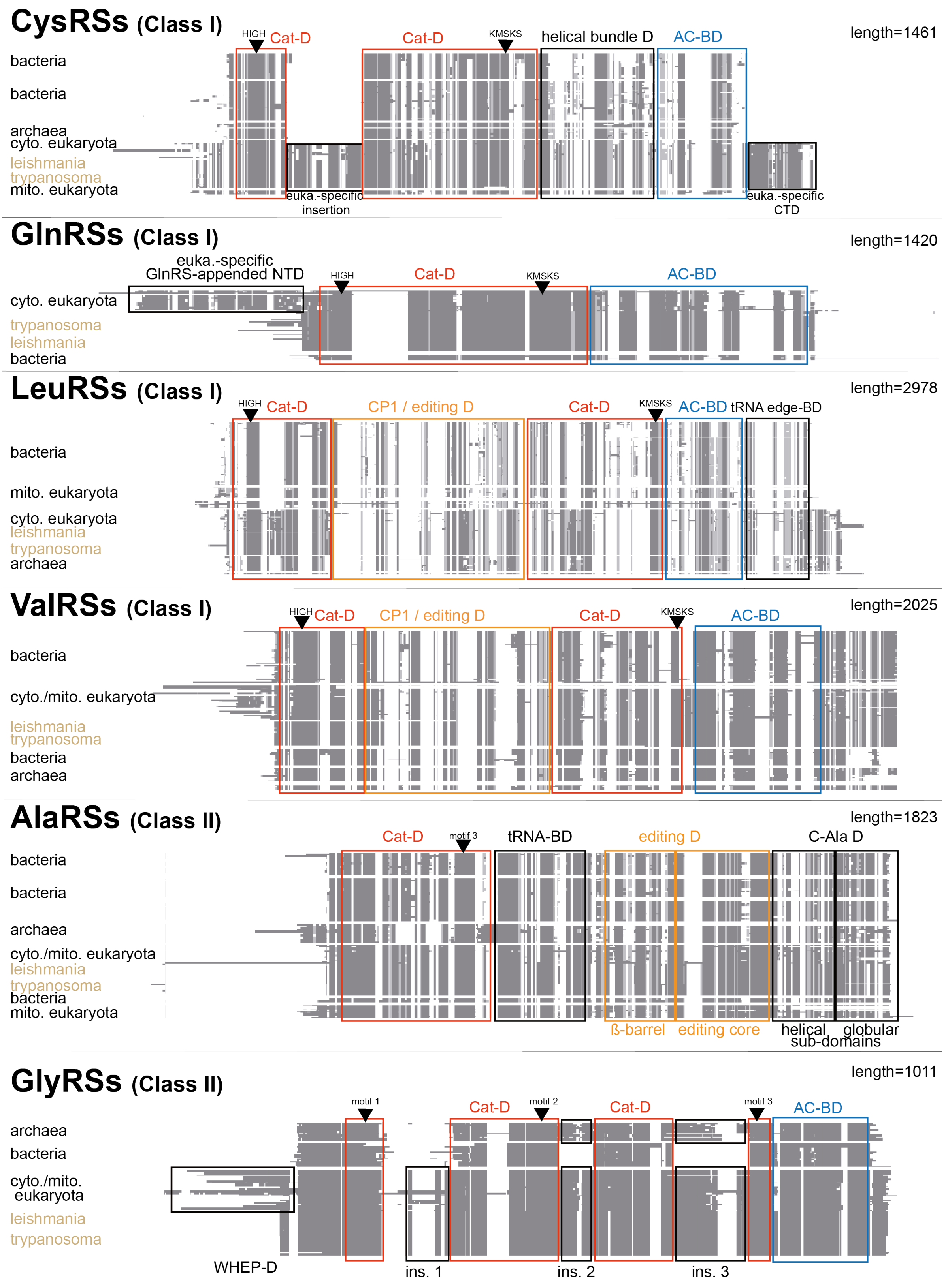

Supplementary Figure S3.

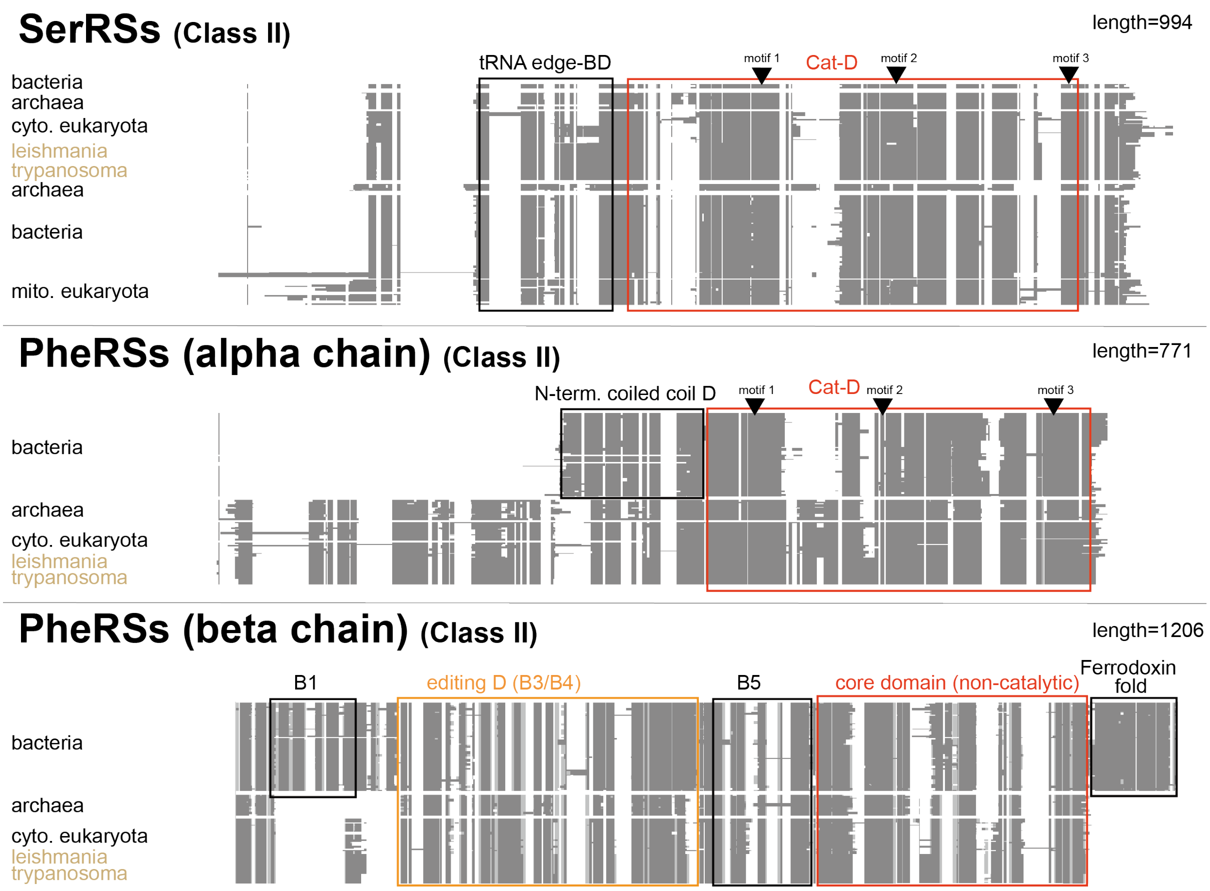

Supporting Figure S4. RNA seq data for *L. tarentolae* GluRS and ThrRS with trypanosomatid-specific extension and insertions. The gray panel is a schematic representation of the coverage obtained after basecalling and alignment of the reads to the reference genome (TriTrypDB-9.0_LtarentolaeParrotTarII_Genome, obtained from the TriTryp database {Aslett, 2010 #8383}). Only reads aligning with aaRS transcripts and containing the spliced leader sequence are selected. An orange arrow represents the leader sequence, and the purple and green boxes represent the 5' and 3' UTRs, respectively.

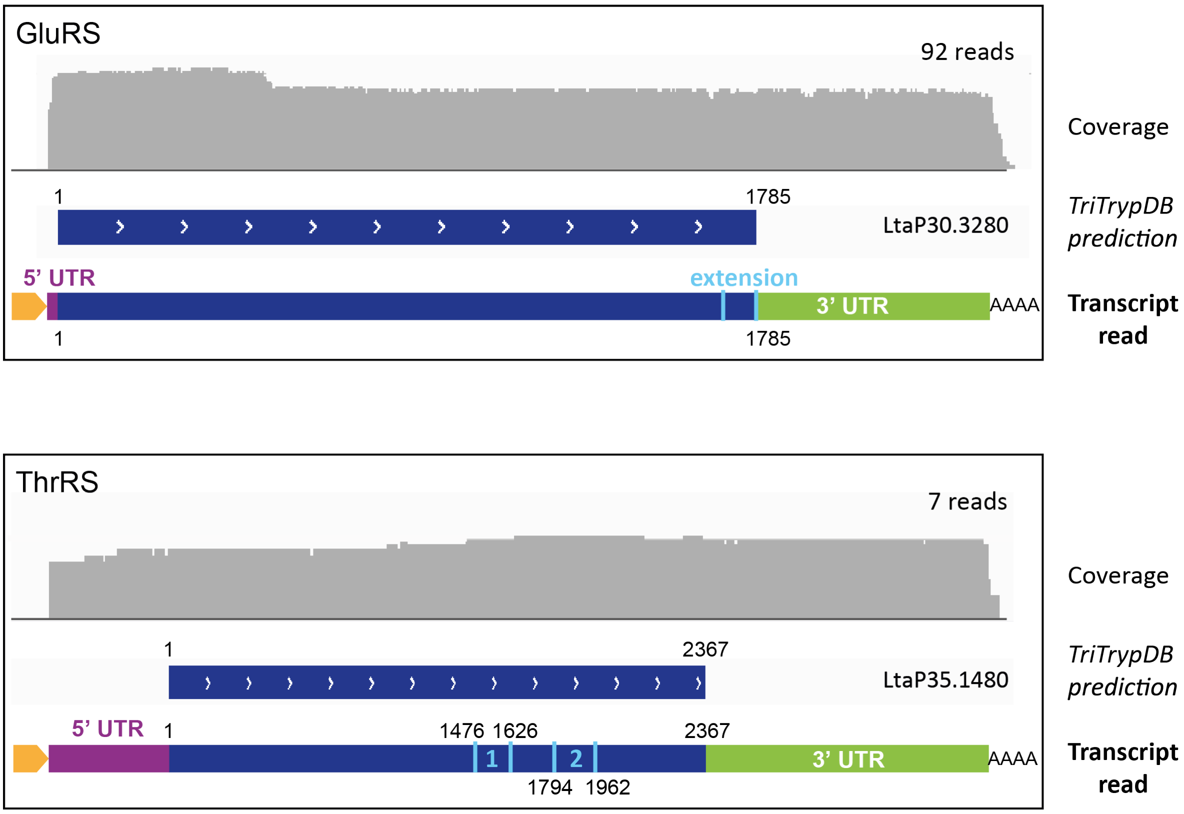

Supporting Figure S5. ProRS sequences from *L. tarentolae*. Regarding the ProRSs, two isoforms are encoded by distinct genes in *Leishmania* species (14). Full-length *L. tarentolae* ProRS sequences are however missing from the TriTryp database. Our RNA-sequencing data allowed us to identify a sequence coding for the mitochondrial isoform (see below). When inserted into the ProRS MSA, it appears that the corresponding protein sequence displays a N-terminal extension of 86 amino acid (see the blue part of the sequence below), similar to the 88 aa N-terminal extension found in the *L. Major* ProRS. This strongly suggests that a similar N-terminal extension (likely the MTS) is present in orthologs from other *leishmania* species.

>ProRS_L_tarentolae_mito (From our RNA Seq data)
MQGRQILSQSHAFFSVLSNSVSFLSSSRLSYRPIVRHSCEQHCLLSYSPRTPSSLTRPAAPVTPTRGYXRSTI
YKQTKSQRIPLDNMSIEDCXGFDEVNALFHELGLDIPILHHDEKATVEEVLDLLQKKGINAAGTKTLFLKSK
KGELVMATAFKSTPTDLKFIQKVTNTKDLRFASNEVLQECLAVVQGCVTPLPLNNNXKEGRPITLLMDSSL
QSSTQPLAFCMCRNDYTAVITFEDLKKYFEKIGYTYKLVDFGAASADAAVTGGSAAPAPKPKKVSSEAAS
AKRGAAAGGAPATAQSGETKLGIAVKREENSSAWYIDVITKAEMIEYYDVSGCYIIRPWAYYVWKCVQR
FLGGQIEKLGVEDCYFPMFVSRGCLEREKDHIEGFAPEVAWVTRAGDTELEQPVAVRPTSETVMYPYYA
KWIRSHRDLPVRLNMWNNVIRWEFSHPTPFIRTREFLWQEGHCAWAKAEECAKEVLDILECYASVYEQ
LLAVPVVRGRKTEKEKFAGGDYTTTVETFIEAVGRGCQGATSHNLGQNFGKMFDIRFQDPENNEQTLIP
WQNSWGLSTRVIGVMIMVHGDNRGMVMPPRVASTQVIIIPVGITKDTTEDARQELLASCRRLEGELRE
GGVRAKCDLRDNYSPGWRFNHWEVKGVPLRVELGPRELAERSLAVAVRHSGARHSVAWDAQTPVAV
AALLEDVQAQMYARAKATMETHRVRVTEWAEFVPTLNRKCLILAPWCGAMECEDQVKKDSAEESKAA
QAQETREDARAPSMGAKTLCIPFEQPEEPAEGHTCICKGCTKPATTWVLFGRSY

Regarding the cytosolic isoform of the ProRS, only a fragment of 486 aa is found in the TriTrypDB (LtaP29.2400) (which corresponds to the green part of the sequence below). Its insertion into the ProRS MSA showed that it corresponds to the C-terminal part of the protein but could not be a functional enzyme since catalytic residues are missing. RNA-sequencing data revealed a candidate sequence, however of incomplete reading (see below). We manually reconstructed a consensus sequence for the *L. tarentolae* cytosolic ProRS (supplementary Figure S4), using the very high sequence conservation observed in the alignment between the cytosolic ProRSs from kinetoplastids.

>ProRS_L_tarentolae_cyto_incomplete (from RNA Seq data)
MSAKDCKGEPELLAXLKELNIXXPTISHGEMHTVEEANRELGRFGTPCMGTKNMFXKSKKGELVLLTAVH
TTKTDMXXIEKAAGVXNLRFAPGEXLSEXLAVVQGCVTPFALINNIXQHNIIVLXDKNLKESPIPXALHPCR
XDKSSLIIFEQLERFLEKIGYXYKLVXFGAPSADAAVTGGSAALAPAAPAPKPKKVSSEAASAKRGAXCWC
ACDCTVWRDEARHRGEARGELFCVVHRCDHEGGDDRVLRRVRVLHYPSLGILRVEVRAAVSXGRSRSLA
WRTATSXCSCPRXCLEREKDHIEGFAPEVAWVTRAXDTELEQPVAVRPTSETVMXPHYAKWIRSHRDLP

VRLNMWNNVIRWEFSHPTPFIRTREFLWQEGTARGRRRRSARRRCWTSWSAMRLCTSSFWRCRWCA
GARRRRRSSLAGTTRRRWRRSLRLLGAGARARRATTWGQNFGKMXDIRFQDPENNEQTLIPWQNSW
GLSTRVIGVMIMVHGDNRGMVMPPRVASTQVIIIPVGITKDTTEDARQELLASCRRLEGELREGGVRRE
VRPARQLQPRVAVQPLGSXXACXLRVELGPRELXERSLAVAVRXSGARHSXAWDAQTPXAVAAALLEXV
QAQMYARAKATMETHRXXVTEWAEFVPTLNRKCLIXAPWCGAMECEDQVKKDSAEESKXAQAQETR
EDARAPSMGAKTLCIPFEQPXXXVEGHTCICKGCXKPATTWVLFGRSY

>ProRS_L_tarentolae_cyto_proposed sequence

MSAKDCKGEPELLAVLKELNIELPTISHGEMHTVEEANRELGRFGTPCMGTKNMFLKSKKGELVLLTAVH
TTKTDMHAIEKAAGTRNLRFAPAEILSENLAVVQGCVTPFALINNIEKHNIIVLLDKNLKESPIPFALHPCRN
DKSSLIIFEQLERFLEKIGYAYKLVDFGAPSADAAVTGGAAAPAPKPKKVSSEAASAKRGGGGAAAAAPAP
APSGETKLGIAARREENFSAWYIDVITKAEMIEYYDVSGCYIIRPWAYYVWKCVQRFLGGQIEKLGVEDCY
FPMFVSRGCLEREKDHIEGFAPEVAWVTRAGDTELEQPVAVRPTSETVMYPYYAKWIRSHRDLPVRLN
MWNNVIRWEFSHPTPFIRTREFLWQEGHCAWAKAEECAKEVLDILECYASVYEQLLAVPVVRGRKTEKE
KFAGGDYTTTVETFIEAVGRGCQGATSHNLGQNFGKMFDIRFQDPENNEQTLIPWQNSWGLSTRVIGV
MIMVHGDNRGMVMPPRVASTQVIIIPVGITKDTTEDARQELLASCRRLEGELREGGVRAKCDLRDNYSP
GWRFNHWEVKGVPLRVELGPRELAERSLAVAVRHSGARHSVAWDAQTPVAVAALLEDVQAQMYARA
KATMETHRVRVTEWAEFVPTLNRKCLILAPWCGAMECEDQVKKDSAEESKAAQAQETREDARAPSMG
AKTLCIPFEQPEPVEGHTCICKGCTKPATTWVLFGRSY

XXX = reconstructed based on conservation on the alignement
XXXX = corresponds to LtaP29.2400 from the TriTrypDB
